# Epigenetic aspects of pancreatic beta cell function in type 2 diabetes

**DOI:** 10.1186/2049-3258-72-S1-K1

**Published:** 2014-06-06

**Authors:** Miriam Cnop

**Affiliations:** 1 Laboratory of Experimental Medicine, Division of Endocrinology, Erasmus Hospital, ULB Center for Diabetes Research, Universite Libre de Bruxelles, Brussels, Belgium

## 

Genetic and environmental factors contribute to the pathogenesis of type 2 diabetes. Epigenetic changes link environmental exposures with potentially heritable disease mechanisms. Pancreatic beta cell failure is central in the pathogenesis of type 2 diabetes. The gold standard for elucidating the underlying mechanisms is the study of human islets of Langerhans. We performed the first comprehensive DNA methylation profiling of islets from type 2 diabetic and non-diabetic donors [1]. We identified differential DNA methylation in 276 CpGs located in the promoters of 254 genes. Methylation changes were not present in circulating blood cells from type 2 diabetic patients. Exposure of islets from non-diabetic donors to high glucose for three days did not induce these methylation changes. An inverse correlation of gene expression and methylation was detected for some genes. Functional annotation of the differentially methylated genes pointed to pathways regulating beta cell dysfunction and death, and this was validated by RNA interference studies. Currently, second generation arrays are being used to interrogate an order of magnitude more CpGs. Taken together, these studies will help to unveil epigenetic disease mechanisms in islets in type 2 diabetes.
